# Patient iPSC-Derived Macrophages to Study Inborn Errors of the IFN-γ Responsive Pathway

**DOI:** 10.3390/cells9020483

**Published:** 2020-02-19

**Authors:** Kathrin Haake, Anna-Lena Neehus, Theresa Buchegger, Mark Philipp Kühnel, Patrick Blank, Friederike Philipp, Carmen Oleaga-Quintas, Ansgar Schulz, Michael Grimley, Ralph Goethe, Danny Jonigk, Ulrich Kalinke, Stéphanie Boisson-Dupuis, Jean-Laurent Casanova, Jacinta Bustamante, Nico Lachmann

**Affiliations:** 1 REBIRTH Cluster of Excellence, Institute of Experimental Hematology, Hannover Medical School (MHH), 30625 Hannover, Germany; haake.kathrin@mh-hannover.de (K.H.);; 2 Laboratory of Human Genetics of Infectious Diseases, Necker Branch, Inserm U1163, Necker Hospital for Sick Children, 75015 Paris, France; 3 Imagine Institute, Paris University, 75015 Paris, France; 4 Institute of Pathology, Hannover Medical School (MHH), 30625 Hannover, Germany; 5 Biomedical Research in Endstage and Obstructive Lung Disease (BREATH), German Center for Lung Research, 30625 Hannover, Germany; 6 Institute for Experimental Infection Research, TWINCORE, Centre for Experimental and Clinical Infection Research, A Joint Venture between The Helmholtz Centre for Infection Research, Braunschweig, and The Hannover Medical School, 30625 Hannover, Germany; 7 Department of Pediatrics, University Medical Center Ulm, 89081 Ulm, Germany; 8 Division of Bone Marrow Transplant and Immune Deficiency, Cincinnati Children’s Hospital Medical Center, Cincinnati, OH 45229, USA; 9 Department of Pediatrics, University of Cincinnati, College of Medicine, Cincinnati, OH 45267, USA; 10 Institute for Microbiology, University of Veterinary Medicine Hannover, 30625 Hannover, Germany; 11 St. Giles Laboratory of Human Genetics of Infectious Diseases, Rockefeller Branch, The Rockefeller University, New York, NY 10065, USA; 12 Howard Hughes Medical Institute, New York, NY 10065, USA; 13 Pediatric Hematology-Immunology and Rheumatology Unit, Necker Hospital for Sick Children, 75015 Paris, France; 14 Study Center for Primary Immunodeficiencies, Necker Hospital for Sick Children, 75015 Paris, France

**Keywords:** hematopoiesis, induced pluripotent stem cells, macrophages, MSMD, mycobacteria, interferon γ

## Abstract

Interferon γ (IFN-γ) was shown to be a macrophage activating factor already in 1984. Consistently, inborn errors of IFN-γ immunity underlie Mendelian Susceptibility to Mycobacterial Disease (MSMD). MSMD is characterized by genetic predisposition to disease caused by weakly virulent mycobacterial species. Paradoxically, macrophages from patients with MSMD were little tested. Here, we report a disease modeling platform for studying IFN-γ related pathologies using macrophages derived from patient specific induced pluripotent stem cells (iPSCs). We used iPSCs from patients with autosomal recessive complete- and partial IFN-γR2 deficiency, partial IFN-γR1 deficiency and complete STAT1 deficiency. Macrophages from all patient iPSCs showed normal morphology and IFN-γ-independent functionality like phagocytic uptake of bioparticles and internalization of cytokines. For the IFN-γ-dependent functionalities, we observed that the deficiencies played out at various stages of the IFN-γ pathway, with the complete IFN-γR2 and complete STAT1 deficient cells showing the most severe phenotypes, in terms of upregulation of surface markers and induction of downstream targets. Although iPSC-derived macrophages with partial IFN-γR1 and IFN-γR2 deficiency still showed residual induction of downstream targets, they did not reduce the mycobacterial growth when challenged with Bacillus Calmette–Guérin. Taken together, we report a disease modeling platform to study the role of macrophages in patients with inborn errors of IFN-γ immunity.

## 1. Introduction

Since their discovery in 2006 [1], induced pluripotent stem cells (iPSCs) have become an extremely valuable tool for drug discovery, autologous cell therapy and disease modeling [2]. Especially for the latter the possibility of generating patient-specific iPSCs carrying a disease-specific mutation has been useful to study genetic diseases and their underlying mechanisms [3]. The feasibility and usefulness of this approach has been highlighted especially for rare diseases, for which patient material is extremely limited. In addition, patient-specific iPSCs are also of great interest for conditions affecting cells that cannot be sampled from patients, i.e., most cells other than erythrocytes, leukocytes and platelets. The generation of iPSCs has been proven for different starting materials and with a wide variety of reprogramming methods [4]. Similarly, the subsequent differentiation in the affected cell type has been shown for many cell types of all three germ layers [5,6,7,8]. Cells of the hematopoietic lineages have been especially in the focus because of their high importance for many diseases, for example, primary immunodeficiencies (PIDs). Of note, also non-hematopoietic cells have been successfully used here to explain susceptibility to herpes simplex virus 1 in the central nervous system [9], influenza pneumonitis [10,11] or viral myocarditis. Cells have been differentiated into megakaryocytes to study thrombocytopenia [12]; erythrocytes to model sickle cell disease [13]; hematopoietic progeny and NK cells to study GATA2 deficiency [14] and macrophages to model such diverse diseases as hereditary pulmonary alveolar proteinosis [15], familial Mediterranean fever [16] and Gaucher disease [17]. Patient-specific macrophages are of high interest, as these cells are important mediators in innate and adaptive immunity and tissue homeostasis and are involved in a variety of functions both in disease and health [18]. Inborn errors affecting these cells can lead to diverse severe diseases as these cells play an important role in many different organs.

Interferon-γ (IFN-γ) is a major macrophage activating factor [19] and plays a pivotal role in the host defense machinery against mycobacterial infections. Indeed, germline mutations in the genes affecting the IFN-γ immunity can cause PIDs like for example Mendelian Susceptibility to Mycobacterial Disease (MSMD). MSMD is characterized by predisposition to disease caused by otherwise only weakly virulent mycobacterial species such as *Mycobacterium bovis* Bacillus Calmette–Guérin (BCG) and various environmental mycobacteria (EM). The prevalence of MSMD is estimated to be in the range of <1/1.000.000 for most etiologies according to orphanet. MSMD is usually diagnosed in childhood, and patients present mainly mycobacterial infections but can also suffer from infections with *Salmonella spp*, fungi, parasites, intra-macrophagic bacteria and even viruses. Of note, patients are also vulnerable to the more virulent *Mycobacterium tuberculosis* (Mtb) [20,21,22]. MSMD can be understood more as an umbrella term for various genotypes that share the core characteristic of susceptibility to mycobacterial disease but that can present more variable clinical and cellular phenotypes [23]. As of now, mutations in 15 genes (*IFNGR1, IFNGR2, STAT1, IL12B, IL12RB1 IL12RB2, IL23R, RORC, IRF8, ISG15, TYK2, JAK1 SPPL2A, CYBB* and *NEMO*) have been described in the context of isolated or syndromic MSMD that cause either a disruption of the production of IFN-γ or of the reaction to IFN-γ [20,23,24,25,26]. Mutations can be autosomal recessive (AR) or dominant (AD) or x-linked (XR) and can cause complete or partial functional impairment [27]. Allelic heterogeneity at several of these loci results in up to 30 different etiologies. The therapy options for MSMD patients relay mainly on antibiotic treatment and IFN-γ substitution. For severe cases of MSMD—mainly presented by patients with AR complete deficiency of the IFN-γ receptor (IFN-γ)—allogenic hematopoietic stem cell transplantation (alloHSCT) remains the only curative treatment option [23,28]. However, this approach is impeded by problems due to recurrent infections and poor engraftment caused by high IFN-γ plasma levels [29,30,31]. While HSCT is mostly necessary for AR complete IFN-γR deficiencies, there also have been two patients reported with particular forms of AR IFN-γR deficiency affecting the first initiation codon, c.2T>A for partial recessive (PR) IFNγR1 deficiency and c.1A>G for IFNγR2 deficiency, who suffered from disseminated mycobacterial infectious diseases requiring aggressive antibiotic therapy and HSCT [26,32].

The discovery of MSMD has highlighted the dependency of anti-mycobacterial activity on IFN-γ immunity; however, the genetic etiology of about half of all MSMD patients remains unknown as of today and the cellular basis remains poorly characterized. Given the low availability of patient material, the limited treatment options and the poorly characterized pathomechanism of MSMD, we wanted to establish a disease modeling platform to study IFN-γ response in macrophages using patient-derived iPSCs. Indeed, it was shown decades ago that IFN-γ can activate macrophages in vitro, thereby directing their anti-microbial mechanisms and up-regulating pathways connected to antigen processing and presentation [33]. Paradoxically, macrophages from patients with MSMD were little tested. As macrophages are the tissue-resident phagocytes and the cells that fail to destroy mycobacteria in MSMD patients, we intended to study their anti-mycobacterial activity and IFN-γ responsiveness in MSMD patient-derived cells. For this purpose, we used material from patients suffering from AR complete- and partial IFN-γR2 deficiency, AR partial IFN-γR1 deficiency and AR complete STAT1 deficiency. Using the various patient-specific iPSC lines, we aimed to gain insights into macrophages and their IFN-γ-independent and IFN-γ-dependent functionality to establish a platform to study IFN-γ pathologies, which could be beneficial for the development of new drugs in the context of MSMD and other related disorders.

## 2. Materials and Methods

### 2.1. Ethical Statement

Human fibroblast samples (c.1A>G *IFNGR2*, c.2T>A *IFNGR,* c.1928insA *STAT11*) were collected after written informed consent of the donor (ID-RCB number 2010-A00650-39) at the Necker Hospital for Sick Children, Paris (France). Human peripheral blood samples (c.705C>A *IFNGR2*) were collected after written informed consent of the donor (IRB number 2012-1156) at the Cincinnati Children’s Hospital Medical Center (USA).

### 2.2. Reprogramming of pPatient Cells into iPSCs and Cultivation of iPSCs

Fibroblasts or peripheral blood was obtained from the patients harboring the *IFNGR2* c.705C>A, *IFNGR2* c.1A>G and *IFNGR1* c.2T>A mutation. Fibroblasts were cultured in low Glucose DMEM supplemented with 10% fetal calf serum (FCS) and 1% penicillin-streptomycin (PS) and were seeded at a density of 3–5 × 10^4^ cells for reprogramming. CD34^+^ cells were isolated from peripheral blood using density centrifugation and magnetic activated cell sorting (MACS) and cultured in StemSpan (#09600 Stem Cell Technologies, Cologne, Germany) supplemented with 2 mM L-glutamine, 1% PS, 100 ng/mL human stem cell factor (#300-07 Peprotech, Hamburg, Germany), 100 ng/mL hFlt3-L (#300-19 Peprotech) and 50 ng/mL human thrombopoietin (#300-18 Peprotech) for 24 h. For reprogramming, a “four-in-one” 3rd generation SIN lentiviral vector containing the original Yamanaka factors was used [34], and fibroblasts or CD34^+^ cells were transduced at a multiplicity of infection (MOI) of 0.3–1 or 10–20, respectively. Cells were reprogrammed in the presence of protamine sulfate, and CD34^+^ cells were placed in a reaction tube on a 360° vertical multi-function rotator (PTR-35, Grant Bio, Cambridge, United Kingdom) at 2 rpm for 6h to ensure continuous mixing with the viral particles. After one day, 0.5 µM valproic acid was added and after 3–5 days, cells were transferred to a murine feeder layer. Cells were grown in half–half medium consisting of the respective culture medium (low Glc DMEM or full StemSpan) and human iPSC medium (knock-out Dulbecco’s modified Eagle medium, 20% knock-out serum replacement, 0.1 mM beta-mercaptoethanol, 1 mM L-glutamine, 1% nonessential amino acids, 1% PS and 10 ng/mL bFGF (#13256029 Invitrogen, Schwerte, Germany). After initial colony appearance, medium was switched to only human iPSC medium and colonies were picked to establish clones. Cells were maintained on a murine feeder layer and split every 7–10 days using 2 mg/mL collagenase IV (#17104-019 Gibco Schwerte, Germany).

### 2.3. Genotyping

gDNA was isolated using the GenElute Mammalian Genomic DNA Miniprep Kit (#G1N70 Sigma, Hamburg, Germany) following the manufacturer’s instructions. gDNA (100 ng) was amplified by PCR with Phusion^®^ High-Fidelity DNA polymerase (#M0530S NEB, Frankfurt, Germany) according to manufacturer’s instructions, and the following primers have been used: *IFNGR2* c.1A>G fwd: CTGCTGCTCGGGAAGAGG rev: TGATCTGAGCACTCCGC ATA, *IFNGR2* c.705C>A fwd: CCAGCCAGTGACCCACTAAA rev: AAATGGGCAAG TCCC TCTACC, *IFNGR1* c.2T>A fwd: CTCAAATTCCTCCCACACCCA rev. CAACCGACGAGTTCAAACCAC, *STAT1* c.1928insA fwd: CACATCATTGAAGAT GCAGGCT rev: CACCTGCACTGAGTTTATGCC.

### 2.4. Immunofluorescence Staining

Immunofluorescence staining was carried out according to standard protocols. Primary antibodies used were anti-OCT4 (#sc5279 Santa-Cruz, Heidelberg, Germany, 1:200), anti-NANOG (#sc293121 Santa-Cruz, 1:200), anti-TRA1-60 (MAB4360 Millipore, Darmstadt, Germany, 1:250) and anti-SSEA-4 (MAB4304 Millipore; 1:250). Secondary antibodies used were goat anti-mouse IgG Alexa-546 (#A-11030 Invitrogen, Schwerte, Germany) and goat anti-mouse IgM Alexa-488 (#A-21042 Invitrogen). Nuclei were co-stained with 4,6-diamidino-2-phenylindole DAPI (1:2000). Alkaline Phosphatase staining was performed using the Alkaline Phosphatase Staining Kit II (#00-0055 Stemgent, Glasgow, United Kingdom) according to the manufacturer’s instructions.

### 2.5. Teratoma Formation

hiPSC were incubated with 10 µM Y-27632 (Leibniz University Hannover) one hour before detachment with 1 mg/mL dispase 1 (Roche, Mannheim, Germany). For teratoma induction, 5 × 10^6^ cells were suspended in 100 µl hiPSC medium with 20 µM Y-27632 and mixed with 100 µL Matrigel (#354277 Corning, Kaiserslautern, Germany). Cells were subcutaneously injected into the flanks of immunodeficient mice (NOD.Cg-Prkdcscid Il2rgtm1Wjl/SzJ-background). After teratomas reached 1.5 cm in diameter, mice were sacrificed and teratoma tissue was fixed with 4% phosphate-buffered formaldehyde. Then, tissue was embedded in paraffin blocks. For histology, teratoma specimens were stained with hematoxylin and eosin to determine germ layer descendants.

### 2.6. Generation of iPSC-Derived Macrophages

iPSC-derived macrophages were generated as described previously [35]. In brief, iPSCs were first cultured as described above. After 3–5 days, bFGF was omitted from the culture media and embryoid bodies were formed on an orbital shaker at 80rpm. After 5 days, EBs were manually selected and transferred into differentiation media (X-Vivo (#BE02-060F Lonza, Cologne, Germany) supplemented with 1% PS, 1 mM l-glutamine, 0,05 mM β-mercaptoethanol, 50 ng/L M-CSF and 25 ng/mL IL-3). Media was changed on a weekly basis, and after 2 weeks, monocytic cells could be harvested from the supernatant and terminally differentiated in RPMI supplemented with 1% FCS, 1% PS and 50 ng/mL M-CSF (Standard macrophage medium).

### 2.7. Cytospins

For cytospins 3–5 × 10^4^ cells were centrifuged on object slides at 600× *g* for 7 min. Next, dried slides were stained for 5 min in May–Grünwald staining solution (0.25% (*w*/*v*) in methanol), followed by 20 min in 5% of Giemsa azur-eosin-methylene blue solution (0.4% (*w*/*v*) in Methanol, working solution was 0.02%) and washed in aqua dest.

### 2.8. Flow Cytometric Analysis

For flow cytometric analysis 1–2 × 10^5^ cells were stained in PBS with 2% FSC and 2 mM EDTA for 45 min on ice. For upregulation of IFN-γ controlled surface markers, cells were stimulated with 25 ng/mL IFN-γ (#300-02 Peprotech) for 24 h before flow cytometric analysis. For analysis of pluripotency markers cells were harvested using TrypLE Express (#12605028 Gibco) to obtain single cells. The following antibodies were used: SSEA4 (#560126 BD Bioscience, Heidelberg, Germany), TRA-1-60 (#330609 Biolegend, Koblenz, Germany), CD14 (#12-0149-41 eBioscience, Schwerte, Germany), CD11b (#17-0118-41eBioscience), CD45 (#17-0459-42 eBioscience), CD163 (#12-1639-41 eBioscience), CD282 (#12-9922-41 eBioscience), CD64 (#11-0649 eBioscience), CD38 (#17-0389-41 eBioscience), HLA-DR (#307609 Biolegend), IFNGR1 (#12-1199 eBioscience), IFNGR2 (#RDAF773 R&D, Abingdon, United Kingdom). Respective isotype controls were used. Cells were measured with a Cytoflex S (Beckman Coulter, Krefeld, Germany) and analyzed using FlowJo10.

### 2.9. qRT-PCR

RNA was extracted using Trizol (Bioline #BIO-38032, BioCat, Heidelberg, Germany) and cDNA was synthesized using RevertAid reverse transcriptase (#EP0442 Fermentas, Schwerte, Germany) and random hexamer primers (#SO142 Fermentas). For qRT-PCR analysis TaqMan Universal Master Mix II (#4440038 Applied Biosystems, Schwerte, Germany for pluripotency genes) or SYBR Green PCR Master Mix (4309155 Applied Biosystems, Schwerte, Germany, for genes upregulated after IFN-γ stimulation) was used according to the manufacturer’s instructions. For detecting genes upregulated by IFN-γ, cells were stimulated with 25 ng/mL IFN-γ for 24h before RNA was isolated. The following primers were used for SYBR qR-TPCR: GAPDH (#QT01192646 Qiagen, Hilden, Germany), *IRF1* (#QT00494536 Qiagen), *CCL2* (#QT00212730 Qiagen), *CCL4* (#QT01008070 Qiagen), *CXCL10* [36]. The following primers were used for TaqMan qRT-PCR: *ACTIN* (#Hs99999903_m1 Thermo Fisher, Schwerte, Germany), *NANOG* (#Hs02387400_g1 Thermo Fisher), *OCT4* (#Hs0300511g1 Thermo Fisher), *SOX2* (#Hs01053029s1 Thermo Fisher).

### 2.10. Western Blot

For Western blot analysis, cells were starved in X-Vivo supplemented with 1% PS, 1 mM l-glutamine and 0.05 mM ß-mercaptoethanol for 24 h and stimulated with 25 ng/mL or 100 ng/mL IFN-γ for 30 min. Afterwards, proteins were isolated using RIPA-buffer with phosphatase inhibitor. Proteins were separated on an 8% SDS-PAGE and blotted on PVDF. The following antibodies were used: pSTAT1 (#9167S Cell Signaling, Frankfurt, Germany), STAT1 (#14994S Cell Signaling), Tubulin (#T9026 Sigma).

### 2.11. GM-CSF Clearance

For measurement of GM-CSF uptake, 1 × 10^5^ cells were seeded in a 48-well plate in standard macrophage medium. After 24 h, medium was replaced with medium containing 1 ng/mL GM-CSF and supernatant samples were collected at time points 0, 24 and 48 h. GM-CSF levels were measured via ELISA (#88-8337-22 eBioscience) following the manufacturer’s protocol.

### 2.12. Phagocytosis Assay

For measuring phagocytic uptake, 1–2 × 10^5^ cells were seeded in 24-well plates in standard macrophage medium. After 24 h, media was replaced with standard macrophage media supplemented with 50 mM HEPES, and cells were incubated for 6 h with fluorescent labeled *Escherichia coli* bioparticles (#P35361 Thermo Fisher) at 37 °C. Bioparticles are pH-sensitive and become brightly fluorescent after uptake into the phagosome. After washing of the cells, uptake of bioparticles was measured using a Cytoflex S (Beckmann Coulter) and analyzed using FlowJo10. Microscopic pictures were taken using an Olympus IX71.

### 2.13. Mycobacterial Infection Assay

Mycobacterial killing by macrophages was determined as previously described [37]. In brief, BCG cultures were centrifuged, washed and resuspended in DMEM medium to achieve an OD_600_ of 0.1. Single cell suspension was ensured via ultrasonic treatment of solution and low speed centrifugation at 120 G for 2 min. For the infection iPSC-derived macrophages were seeded on a 12-well plate at a density of 2 × 10^5^ cells per well and pre-stimulated with 25 ng/mL IFN-γ 24 h prior to infection. Cells were infected with a multiplicity of infection (MOI) of 5:1 (corresponding to an OD_600_ of 0.1) for 1 h. The cells were treated with 10 µg/mL gentamicin 1 h post-infection. Moreover, 1 h and 24 h post-infection, the cells were washed twice with PBS, scraped of the plates and resuspended in 1 mL 1% Nonident P40 in PBS. Cells were disrupted by passing them at least 10 times through a 24-gauge needle. Afterwards tenfold serial dilutions of the homogenates were plated on Middlebrook 7H10 agar plates. Plates were incubated for up to 2 weeks at 37 °C until colony-forming units (CFU) were counted.

### 2.14. Superoxide Anion Assay

To estimate the production of reactive oxygen species (ROS) after IFN-γ stimulation the Superoxide Anion Assay (CS1000-1KT, Sigma) has been used according to the manufacturer’s instructions with the following changes: Cells have been pre-stimulated for 24 h with 50 ng/mL IFN-γ, and fresh IFN-γ has been added during the assay; 2 × 10^5^ cells have been used per condition, and luminescence has been measured after 10 min.

### 2.15. Microarray Analysis

For microarray analysis macrophages have been stimulated with 100 ng/mL IFN-γ for 2h or left as unstimulated controls. RNA was isolated using the RNeasy Micro Kit (#74004, Qiagen) according to the manufacturer’s instructions. RNA (130 ng) has been used per sample to analyze transcriptome changes after IFN-γ stimulation. The microarray analysis was performed as described before [38]. The microarray used in this study is a version of the Whole Human Genome Oligo Microarray 4 × 44K v2 (Design ID 026652, Agilent Technologies), which was developed by the Research Core Unit Genomics (RCUG) of Hannover Medical School under the name ‘026652QM_RCUG_HomoSapiens’ (Design ID 084555). The microarray was designed with the Agilent eArray portal using a 1 × 1M design format for mRNA expression as a template. Non-control probes have been printed five times within a region making up a total of 181,560 features (170 columns × 1068 rows). Four of these regions were combined in one 1M region resulting in four microarray fields per slide that can be hybridized individually (Customer Specified Feature Layout). Control probes needed were automatically determined by eArray using the default settings. Cy3-labeled cRNA was synthesized in ¾ reaction volumes using the ‘Low Input Quick Amp Labeling Kit One Color’ (#5190-2305, Agilent Technologies) following the manufacturer’s instructions. The ‘One-Color Microarray-Based gene Expression Analysis Low Input Quick Amp Labeling Protocol V6.7′ was used for cRNA fragmentation, hybridization and washing, with the change that 2500 ng of labeled cRNA was used for hybridization. Slides were scanned on an Agilent Micro Array Scanner G2565CA with a pixel resolution of 3 µm and a bit depth of 20. The ‘Feature Extraction Software V10.7.3.1′ was used for data extraction using the default extraction protocol ‘GE1_107_Sep09.xml’. On-Chip replicates (Quintuplicates) were averaged by using the geometric mean of the processed intensity values of the green channel (gProcessedSignal = *gPS*). Features were excluded from averaging if they were (1) manually flagged, (2) identified as outliers by the software, (3) lay outside the’1.42 × interquartile range’ considering the normalized *gPS* distribution or (4) showed a variation coefficient of pixel intensities per feature that exceeded 0.5. Afterwards averaged *gPS* values were normalized by global linear scaling: *gPS* values of each sample were multiplied by an array-specific scaling factor. This factor was calculated by dividing a ‘reference 75th percentile value’ (1500 for the series) by the 75th percentile (Array *i* in the formula). Normalized *gPS* were calculated with the following formula:

*normalized gPS_Array i_ = gPS_Array i_ x (1500/75^th^ Percentile_Array i_)* (1)

A lower intensity threshold was defined based on the intensity distribution of all negative control features, which was fixed at 15 normalized *gPS* units. Measurements that fell below this value were substituted by the surrogate value of 15. Genes for which all values were below the cut-off were removed from the analysis. Fold change between stimulated and unstimulated samples was calculated by dividing the averages of each, and significance was assessed via T-test. Heatmap visualization was performed using ‘Cluster 3.0’, and ‘Java Treeview’. Genes were centered and normalized with standard settings. Hierarchical clustering was performed with centroid linkage and standard settings. PCA visualization was performed using the ‘ClustVis’ online tool (https://biit.cs.ut.ee/clustvis/) and WikiPathways analysis was performed using Enrichr (http://amp.pharm.mssm.edu/Enrichr/).

### 2.16. Statistical Analysis

Statistical analysis and visualization of results were performed using GraphPad Prism 8.3.1. Statistical tests are indicated in the respective figure legends. * *p* ≤ 0.05 ** *p* ≤ 0.01 *** *p* ≤ 0.001.

## 3. Results

### 3.1. Patient iPSC Lines Harboring Different Mutations Affecting IFN-γ Response

**Figure 1:**
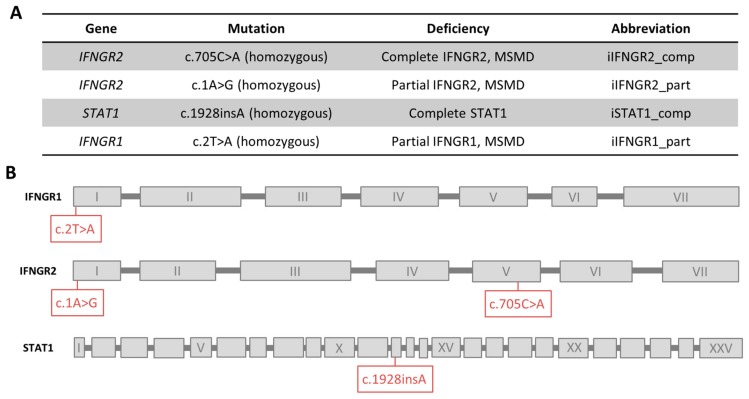
Overview of patients and their mutations. (**A**) Overview of mutations of the patients, the connected deficiency and the abbreviation used in this study. (**B**) Schematic overview of the location of each mutation in the genes. Exons are not to scale.

For the establishment of the disease modeling platform, we received material from three unrelated MSMD patients. The first patient has an AR complete IFNγR2 deficiency due to a mutation (c.705C>A, p.T235*) in the fourth exon of *IFNGR2* that leads to a premature stop codon and no surface expression of the receptor chain [32]. The second patient has an AR partial IFNγR2 deficiency due to start codon mutation (c.1A>G, predicted p.M1V) that possibly leads to lower expression of a truncated but still functional protein [26]. The third patient has an AR partial IFNγR1 deficiency also due to a start codon mutation (c.2T>A, predicted p.M1K) that possibly leads to residual expression of a functional protein [39]. As a further control, we included an already existing iPSC line from a patient that has a AR complete STAT1 deficiency due to a frameshift mutation (c.1928insA) [40]. An overview of the respective patients and their mutations is shown in Figure 1A,B.

**Figure 2:**
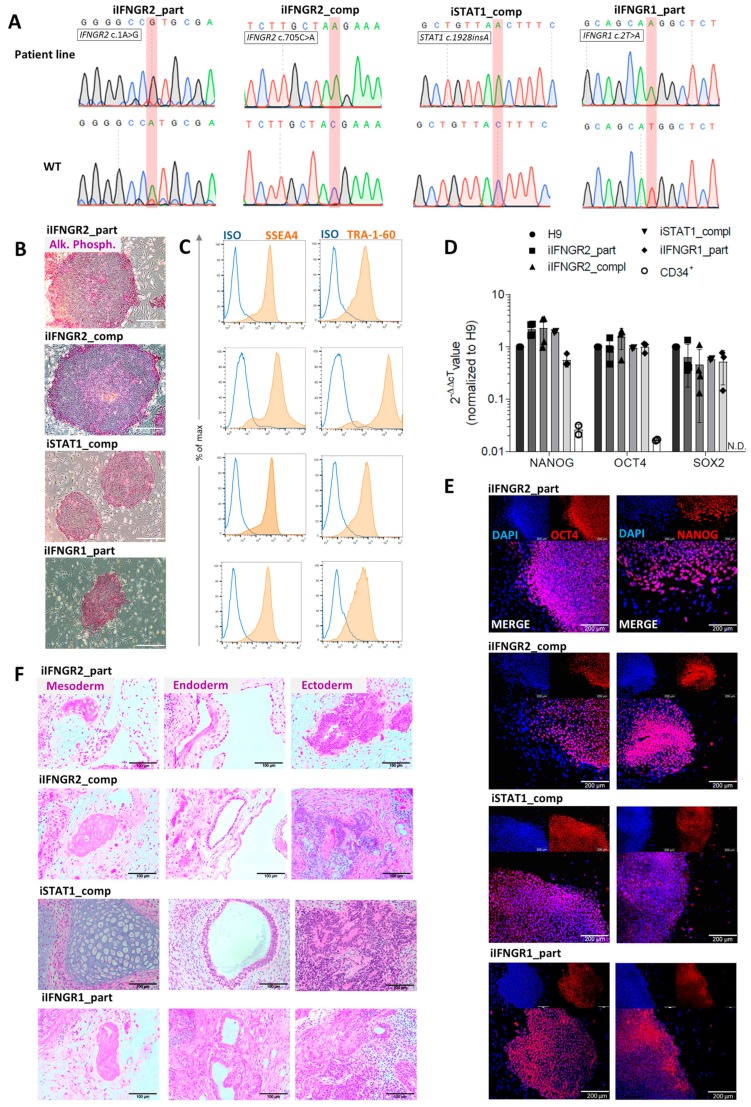
Characterization of patient specific iPSCs. (**A**) Sequence and electropherogram for the patient-specific mutation. Sequencing was performed on the patient line (top) and a healthy iPSC line (bottom) as wild type (WT) control. The mutated nucleotide or insertion is highlighted in red. (**B**) Staining for alkaline phosphatase (AP) activity of patient iPSCs. Scale bar = 200 µm. (**C**) Representative flow cytometric analysis of SSEA4 (left) and TRA-1-60 (right) expression on patient iPSCs. Blue: Isotype. Orange: Marker. (**D**) qRT-PCR analysis of endogenous *NANOG, OCT4* and *SOX2* expression of patient iPSCs normalized to H9 control cells. *ACTIN* was used as a housekeeping gene and CD34^+^ cells as negative control. N.D. = not detectable (*n* = 3 biological replicates, mean ± SD). (**E**) Representative images of cells of the three germ layers after teratoma formation after injection of patient iPSCs into immunodeficient mice. Scale bar = 100 µm. (**F**) Representative immunofluorescent staining for OCT4 (left) and NANOG (right) in patient iPSCs co-stained with DAPI. Scale bar = 200 µm.

iPSCs were generated either from primary fibroblasts (iIFNGR2_part and iIFNGR1_part) or CD34^+^ cells isolated from peripheral blood (iIFNGR2_comp) as starting material. For the reprogramming a “Four-in-One”, 3rd generation SIN lentiviral vector containing the original four Yamanaka factors was used [34]. After 5–7 days, reprogrammed cells were transferred to a murine feeder layer. After another 7–14 days, colonies appeared and subclones were established. Genotypic analysis of established iPSCs confirmed the patient-specific mutation, which was absent in a healthy control iPSC line (hCD43iPSC16) [15,35,41] (Figure 2A). Of note, iPSCs were also verified for the mutations of all patients (*IFNGR2* c.705C>A, *IFNGR2* c.1A>G, *IFNGR1* c.2T>A, *STAT1* c.1928insA) and were negative for the other patient-specific mutations (Figure S1A). In addition, established patient specific iPSC lines showed typical morphology, stained positive for alkaline phosphatase (AP) activity (Figure 2B) and showed upregulation of the common pluripotency markers SSEA4 and TRA-1-60 in flow cytometric analysis (Figure 2C) and immunofluorescence (Figure S1B). Moreover, they showed reactivation of endogenous *NANOG, SOX2* and *OCT4* expression (Figure 2D,E). Pluripotency was further proven by the ability of all clones to differentiate into cells of all three germ layers as part of a teratoma formation after injection into immunodeficient mice (Figure 2F).

### 3.2. Differentiation of Patient iPSC Lines Results in Functional Macrophages

**Figure 3:**
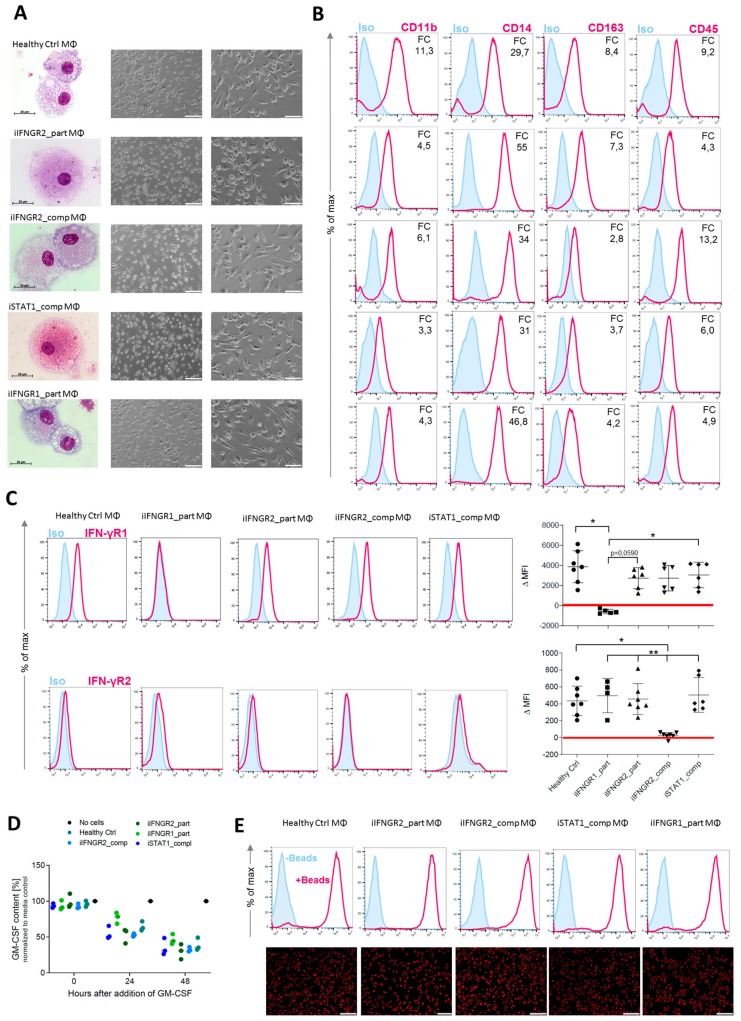
Generation of patient specific iPSC-derived macrophages. Patient iPSCs have been differentiated into macrophages and compared to macrophages from a healthy iPSC line (hCD34_iPSC16). (**A**) Microscopic analysis of patient iPSCs in cytospin images after Pappenheim staining (left, scale bar = 20 µm) or in brightfield images (middle scale bar 200 µm, right scale bar = 100 µm). (**B**) Representative flow cytometric analysis of CD11b, CD14, CD163 and CD45 expression on patient iPSCs and healthy macrophages of two independent experiments. Blue: Isotype. Pink: Surface marker. FC = fold change of the median fluorescent intensity. (**C**) Flow cytometric analysis of IFN-γR1 (top) and IFN-γR2 (bottom) expression on healthy and patient iPSC-derived macrophages. Blue: Isotype. Pink: Surface marker. Expression has been quantified by plotting the difference of the median fluorescent intensity (ΔMFI). Each dot represents macrophages from an independent harvest and from at least three independent differentiations (*n* = 4–7, mean ± SD, Kruskal–Wallis with Dunn’s multiple comparison). Red line shows ΔMFI of 0. (**D**) GM-CSF clearance of healthy and patient iPSC-derived macrophages over a time of 48 h. Concentrations have been normalized to control well containing no cells (media only) (*n* = 3, mean ± SD; each dot represents macrophages from an independent harvest and from at least three independent differentiations). (**E**) Representative flow cytometric (top) and microscopic (bottom) analysis of phagocytic uptake of pH-sensitive fluorescent labeled *E. coli* bioparticles of healthy and patient iPSC-derived macrophages of two independent experiments; scale bar = 500 µm.

To better study IFN-γ-immunity, we subjected the different patient-derived iPSCs and the healthy control line (hCD34_iPSC16) to an already established hematopoietic differentiation protocol to generate macrophages [35]. All iPSC lines were able to form myeloid cell forming complexes (MCFCs) and started to continuously produce monocytic cells after approximately 14 days of differentiation. Cells could be harvested on a weekly basis and in similar quantities. After terminal differentiation into macrophages, cells showed typical morphology in brightfield and cytospin images (Figure 3A). Furthermore, macrophages of all patient-derived iPSCs showed a typical surface marker profile of CD45^+^CD11b^+^CD14^+^CD163^+^ (Figure 3B). We also analyzed the cells for their surface expression of IFN-γR1 and IFN-γR2 and found lower or no expression of the receptors in macrophages derived from the patients with an AR complete or partial deficiency. Of note, iIFNGR1_part macrophages showed the lowest difference of the median fluorescence intensity (ΔMFI) for IFN-γR1, while iIFNGR2_comp macrophages showed the lowest ΔMFI for the expression of IFN-γR2 on the cell surface (Figure 3C). Regardless of their mutation, all macrophages showed general macrophage functionality as shown by cytokine uptake and phagocytosis. Here, macrophages were able to uptake GM-CSF from the media (Figure 3D) and phagocytose pH-sensitive fluorescent-labeled *E. coli* bioparticles to a similar extent (Figure 3E). These results indicate that the patient-mutations do not influence the various IFN-γ-independent functionalities tested.

### 3.3. Patient Macrophages Exhibit Impaired IFN-γ Response at Different Stages of the Pathway

**Figure 4:**
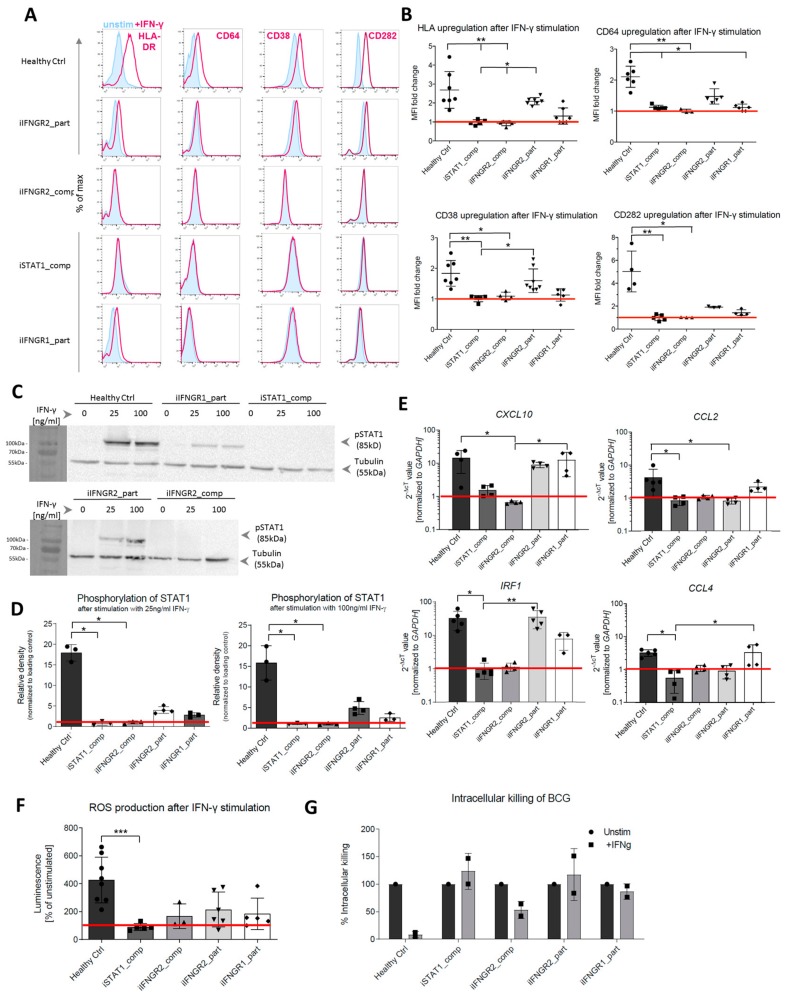
Patient mutations lead to IFN-γ-dependent defects in the iPSC-derived macrophages. (**A**) Representative flow cytometric analysis of surface marker (HLA-DR, CD64, CD38, CD282) upregulation after IFN-γ stimulation in healthy and patient iPSC-derived macrophages. Blue: Isotype. Pink: Surface marker. (**B**) Fold change of median fluorescent intensity (MFI) of HLA-DR, CD64, CD38 and CD282 in healthy and patient iPSC-derived macrophages (*n* = 3–7, mean ± SD; each dot represents macrophages from an independent harvest and from at least three independent differentiations, Kruskal–Wallis with Dunn’s multiple comparison)) (**C**) Representative western blot analysis of STAT1 phosphorylation (pSTAT1) after stimulation with low (25 ng/mL) or high (100 ng/mL) dose of IFN-γ. Tubulin was used as a loading control. The left side of the blot shows the protein size marker. (**D**) Densitometric analysis of STAT1 phosphorylation after IFN-γ stimulation. Values have been normalized to loading control. (*n* = 3, mean ± SD; each dot represents macrophages from an independent harvest and from at least two independent differentiations, Kruskal–Wallis with Dunn’s multiple comparison). (**E**) qRT-PCR analysis of upregulation of downstream targets *IRF1, CXCL10, CCL2* and *CCL4* after IFNγ stimulation. Values have been normalized to GAPDH as housekeeping gene. (*n* = 3–5, mean ± SD; each dot represents macrophages from an independent harvest and from at least two independent differentiations, Kruskal–Wallis with Dunn’s multiple comparison). (**F**) Production of reactive oxygen (ROS) species after IFN-γ stimulation measured via superoxide anion production (*n* = 3–7, mean ± SD; each dot represents macrophages from an independent harvest and from at least two independent differentiations, Kruskal–Wallis with Dunn’s multiple comparison). (**G**) Intracellular killing of BCG in unstimulated and stimulated samples. Intracellular killing of BCG was calculated in percent of killing 24 h after infection compared to 15 min after infection. BCG load of cells was evaluated by plating of macrophage cell suspension on Middlebrook 7H10 agar plates (*n* = 2, mean ± SEM; each dot represents macrophages from an independent harvest).

As a next step, we analyzed the consequences of the AR IFN-γR1/2 or STAT1 deficiency in fully differentiated macrophages to assess the impact on the response to IFN-γ. First, we performed flow cytometric analysis of the upregulation of common surface markers that are known to be induced by IFN-γ stimulation. Here, upon stimulation with IFN-γ, HLA-DR, CD64, CD38 and CD282 showed increased surface marker expression in healthy control macrophages, whereas no upregulation in macrophages derived from patient-specific iPSCs could be observed (Figure 4A). In addition to the initial assessment of surface marker expression, we further quantified the upregulation and analyzed the fold change of the MFI between stimulated and unstimulated samples (Figure 4B). Irrespective of the surface marker analyzed, healthy control macrophages showed the highest fold change, whereas the iIFNGR2_part macrophages showed a reduced induction. Of note, the iIFNGR1_part macrophages showed only residual induction. In clear contrast, no upregulation could be detected in iIFNGR2_comp and iSTAT1_comp macrophages, suggesting an impaired downstream signaling pathway. Moreover, the stimulation with IFN-γ itself did not have any negative effect on cell viability (Figure S1E). To underline this observation, we next analyzed the phosphorylation of STAT1 as a major downstream effector molecule of the IFN-γR pathway. Healthy control macrophages phosphorylated STAT1 after stimulation with a low (25 ng/mL) and high (100 ng/mL) dose of IFN-γ. In contrast, both the iIFNGR1_part and iIFNGR2_part macrophages showed a clear phosphorylation but to a much lesser degree, while no phosphorylation could be detected for the iSTAT1_comp and iIFNGR2_comp macrophages (Figure 4C). In line with these observations, densitometric analysis also showed that the macrophages with the partial deficiency showed a phosphorylation that is much lower than the healthy control macrophages (Figure 4D). Of note, iSTAT1_comp cells showed no expression of STAT1 as expected, whereas the other lines showed normal STAT1 expression (Figure S1C). To further asses the impaired IFN-γR response, we also analyzed the upregulation of downstream target genes of the IFN-γ immunity. Here, *CXCL10* and *IRF1* showed a clear upregulation in healthy control macrophages after IFN-γ stimulation, but also macrophages with a partial deficiency showed a similar upregulation, whereas the AR complete IFN-γR2 and STAT1 deficiencies showed no upregulation. For *CCL2* and *CCL4* healthy control macrophages showed a slight upregulation, as well as the iIFNGR2_part macrophages, whereas the other cells showed no induction (Figure 4E). While IFN-γ is involved in the activation of macrophages and pathogen clearance, we further assessed the functionality of macrophages after IFN-γ stimulation in the context of reactive oxygen species (ROS) production. Similar to the assays before, healthy macrophages showed a strong ROS production post IFN-γ stimulation, while iIFNGR2_part macrophages showed only a slight ROS production. A slight ROS production could however also be observed in iIFNGR1_part and iIFNGR2_comp macrophages and only iSTAT1_comp macrophages showed no production (Figure 4F). As a final functional assessment of patient specific iPSC, we challenged iPSC-derived macrophages with *Mycobacterium bovis* Bacillus Calmette–Guérin (BCG) as an MSMD-relevant pathogen. Following co-culture experiments with BCG, we could observe a reduced growth of BCG for the healthy control macrophages, whereas no to little clearance of BCG was observed for macrophages derived from the various patient specific iPSCs lines (Figure 4G).

### 3.4. Macrophages with AR Complete IFN-γR2 Deficiency Show Dysregulation on Transcriptomic Level

**Figure 5:**
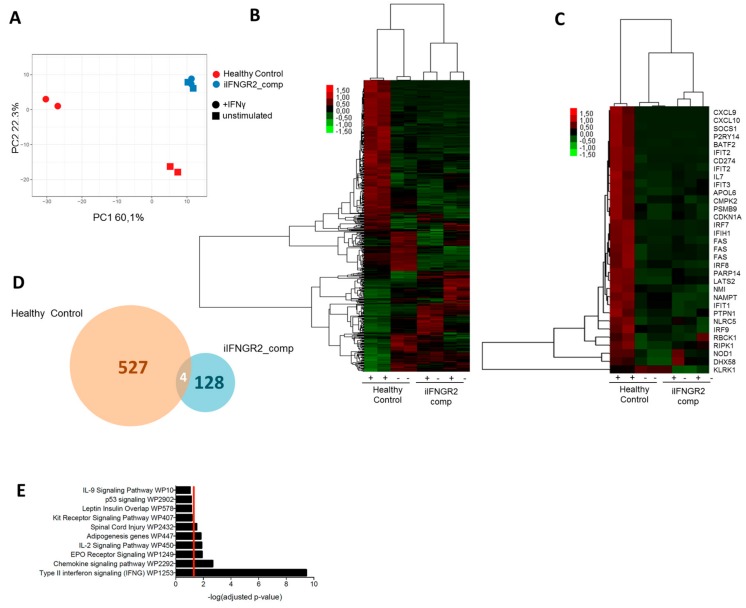
Transcriptional analysis shows differential gene expression in healthy and patient macrophages. (**A**) Principal component analysis (PCA) of unstimulated and stimulated samples of healthy control and iINFGR2_comp macrophages. PCA was performed on all genes significantly changes (*p* ≤ 0.05) in healthy control macrophages. (**B**) Heatmap of all genes significantly up- or downregulated between stimulated and unstimulated healthy control macrophages compared to the other groups. (**C**) Genes of the hallmark IFN-γ response group (M5913) of the molecular signature database (MSigDB) and their expression in healthy control and iIFNGR2_comp macrophages. (**D**) Comparison of genes significantly up- or downregulated (*p* ≤ 0.05) in stimulated compared to unstimulated healthy control macrophages and stimulated compared to unstimulated iIFNGR2_comp macrophages. (**E**) Genes that were significantly up- or downregulated (*p* ≤ 0.05 in stimulated healthy control macrophages compared to the other groups were analyzed via gProfiler for enriched WikiPathway groups. The top 10 groups are shown.

To assess the difference in IFN-γ response of deficient macrophages on a broader scale, we performed microarray analysis of stimulated and unstimulated samples of healthy control and iIFNGR2_comp macrophages. Of note, we used the genetic etiology of complete AR IFN-γR2 deficiency as these showed the most severe cellular and clinical phenotype. Samples of stimulated and unstimulated healthy macrophages clustered apart from each other after principal component analysis, whereas samples of iIFNGR2_comp macrophages clustered together regardless of IFN-γ stimulation (Figure 5A). Heatmap visualization of genes also showed strong gene up/downregulation in stimulated healthy control macrophages compared to unstimulated samples, which was absent in IFNγR2 complete deficient macrophages (Figure 5B). Looking at genes of the hallmark to IFN-γ response category (M5913) of the molecular signature database (MSigDB), many were highly upregulated in the healthy control but showed no change in the iIFNGR2_comp macrophages (Figure 5C). In total, there were 527 genes significantly (*p* ≤ 0.05) up- or down-regulated, whereas only 128 genes changed significantly in the iIFNGR2_comp macrophages, four of which were shared (Figure 5D). It should be noted here that of these genes, none changed significantly with a fold change of two or higher, whereas 133 genes have a fold change above two for the healthy control macrophages. The significantly upregulated genes in healthy macrophages were significantly enriched for IFN-γ signaling in a WikiPathway analysis (Figure 5E), whereas iIFNGR2_comp macrophages were not enriched for any pathways (Figure S1D).

### 3.5. Summary

Given the low availability of patient material, the limited treatment options and the poorly characterized pathomechanism of MSMD we have here established a disease modeling platform to study IFN-γ-related pathologies in macrophages using patient-derived iPSCs. For this, we used material from patients suffering from complete and partial IFN-γR2 deficiency, partial IFN-γR1 deficiency and complete AR STAT1 deficiency. We could show that iPSC-derived macrophages from these patients exhibit varying levels of response to IFN-γ and their deficiencies play out at different stages of the pathway. This highlights the suitability of this platform to study IFN-γ signaling and possibly also screen for new therapy forms for MSMD patients and others.

## 4. Discussion

In the present study, we established a disease modeling platform for IFN-γ related pathologies—mainly MSMD—based on patient iPSC-derived macrophages with a variety of mutations. IFN-γ is a major macrophage activating factor and plays a major role in the pathophysiology of MSMD and other disease entities. After establishing patient-specific iPSCs, we subjected established iPSC-clones to a hematopoietic differentiation protocol to generate patient specific macrophages. We previously highlighted the role of macrophages in MSMD [42] and also the feasibility of using limited patient samples to recapitulate the phenotype in vitro [43]. As PBMC-derived monocytes may be difficult to obtain and current cell systems to study the MSMD pathophysiology have certain shortcomings, we emphasize the MSMD iPSC-macrophage model as a new tool for disease modelling and drug screening. Irrespective of the patient background, all cells showed typical macrophage morphology, surface marker profile and functionality. However, differences could be observed in the IFN-γ-dependent functionality. Compared to healthy iPSC-derived macrophages, all patient iPSC-derived macrophages showed deficiencies, although on varying levels. iSTAT1_comp and iIFNGR2_comp macrophages showed no reaction to IFN-γ stimulation and the most severe phenotype, whereas iIFNGR2_part still shows a slight induction and upregulation of relevant target genes and a milder phenotype. In contrast, iIFNGR1_part macrophages showed only a minor, residual reaction to IFN-γ stimulation as seen by residual upregulation of surface markers and STAT1 phosphorylation. Of note, these findings are in line with the clinical phenotypes observed in the respective MSMD patients. For the patient with the IFN-γR2 c.1A>G mutation (iIFNGR2_part) the clinical presentation is relatively mild, which can also be recapitulated in our iPSC-derived macrophages. Although the exact mechanism remains elusive, a recently published manuscript suggests translation IFN-γR2 from alternative (non-AUG) start codons in the signal sequence that still produce a full-length protein but with less efficiency [26]. This would agree with the phenotype of the iPSC-derived macrophages in our study, which also suggests residual activity. Similarly, we could show that the patient iPSC-derived macrophages have a reduced killing ability when challenged with BCG. Notably, MSMD patients can also show susceptibility to other pathogens, and this disease modeling platform could be utilized to study infections with for example *Salmonella* spp., *Mycobacterium* *tuberculosis* or other pathogens. Of note, the use of iPSC-derived macrophages to study cell-pathogen interactions using other pathogens has been previously demonstrated [44,45]. In addition, the use of iPSC-derived macrophages has been proven in drug screening assays to identify new compounds for infections triggered by *Mycobacterium tuberculosis* (Mtb) [46]. Thus, our patient specific iPSC line could serve as an elegant model to investigate not only IFN-γ immunity but also to identify new drugs. With respect to the later, MSMD patients presented in some cases with intracellular bacterial [47,48], fungal [49,50], parasitic [51] or even viral infections [52]. Some of these may share underlying pathomechanism with MSMD, but due to the limited amount of cases and scarcity of patient material, it has not been possible to draw a clear connection so far. The iPSC-based platform here presented would allow for further study of a possible shared pathomechanism. For this, an expansion with more patient lines, possibly also from other diseases, would further improve the presented iPSC platform. In this line, a further expansion in using also other iPSC-derived cells like NK or dendritic cells would help to complete the picture and capture also the other side of the IFN-γ loop. Similarly, also the generation of tissue-specific macrophages (e.g., alveolar macrophages, Kupffer cells or Langerhans cells) could be of interest as well as genetic modification of iPSCs before differentiation. As mentioned before, also a drug screening approach could be conducted to test substances that help clear non-tuberculous mycobacteria (NTMs) or even Mtb. Huang et al., for example, reported that infection of macrophages with Mtb switches their metabolic phenotype and inhibiting fatty acid oxidation via the drug Etomoxir reduced bacterial growth in the cells [53]. In addition, another study could identify that Mtb and BCG have a different effect on the metabolic phenotype of macrophages, with only Mtb causing the reduction of glycolysis and subsequent dependency on fatty acid oxidation [54]. Our platform would allow to study whether this pathogen induced rewiring of the metabolism also occurs in MSMD macrophages and whether it can be rescued. Notably, this kind of intervention would target the cell rather than the pathogen and could circumvent possible resistances. As BCG and other non-tuberculous mycobacteria are also among leading causes of disseminated disease in immunocompromised patients, this would also have implications for other than MSMD patients. Finally, we contemplate the possibility to edit and fix mutations prior to re-introducing phagocytes back into the patient as a novel cell-based treatment approach.

## Figures and Tables

**Figure A1:**
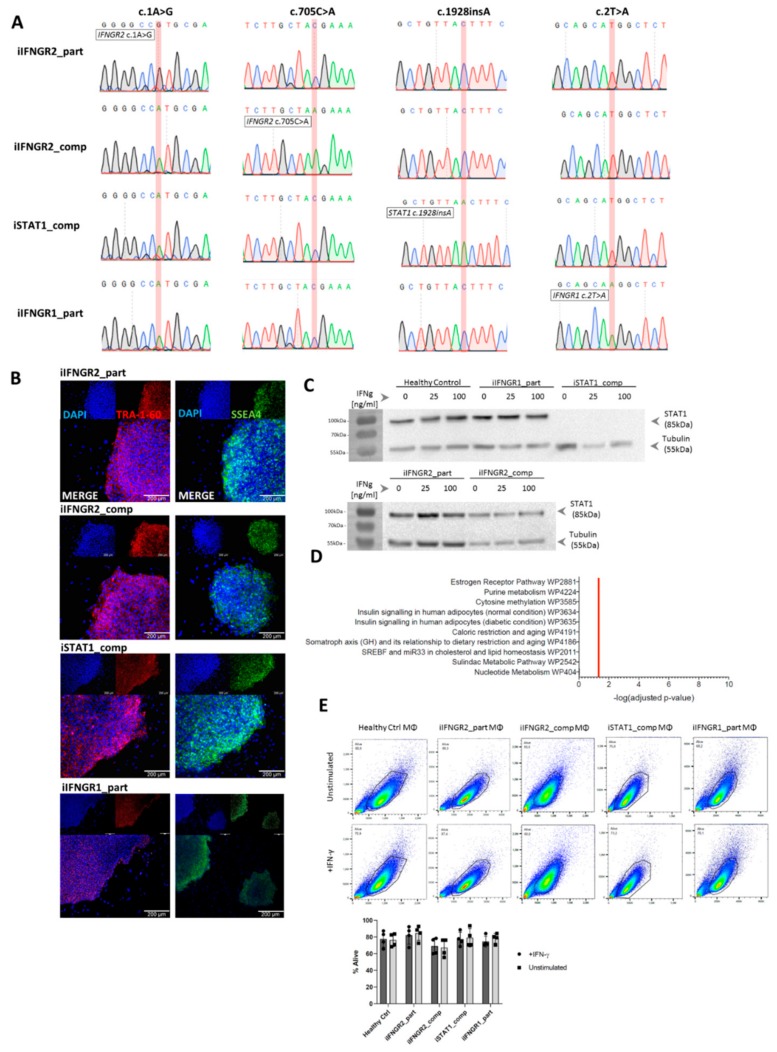
Further analysis of patient-iPSCs and thereof derived macrophages. (**A**) Sequence and electropherogram for the patient-specific mutation. Sequencing was performed on all patient lines for all patient mutations. The mutated nucleotide or insertion is highlighted in red. (**B**) Representative immunofluorescent staining for TRA-1-60 (left) and SSEA4 (right) in patient iPSCs co-stained with DAPI. Scale bar = 200 µm. (**C**) Representative western blot analysis of STAT1 expression in unstimulated samples and samples stimulated with a low (25 ng/mL) or high (100 ng/mL) dose of IFNγ. Tubulin was used as a loading control. (**D**) Genes that were significantly up- or downregulated (*p* ≤ 0.05 in stimulated iIFNGR2_comp macrophages compared to the other groups were analyzed via gProfiler for enriched WikiPathway groups. The top 10 groups are shown. (**E**) Flow cytometry analysis of healthy control and patient-specific iPSC-derived macrophages shows no difference between unstimulated (top row) and stimulated (bottom row) macrophages regarding their viability as evaluated by the FSC/SSC scatter. Quantification was performed for four independent experiments. (*n* = 4; mean ± SD).

## Supplementary Materials

The following are available online at https://www.mdpi.com/2073-4409/9/2/483/s1. Figure S1: Further analysis of patient-iPSCs and thereof derived macrophages (see also Figure A1).
