# Supplementary figures and images for: Patient iPSC-Derived Macrophages to Study Inborn Errors of the IFN-γ Responsive Pathway
Source: Cells. 2020 Feb 19;9(2):483. doi: 10.3390/cells9020483 (PMC7072779; doi:10.3390/cells9020483)

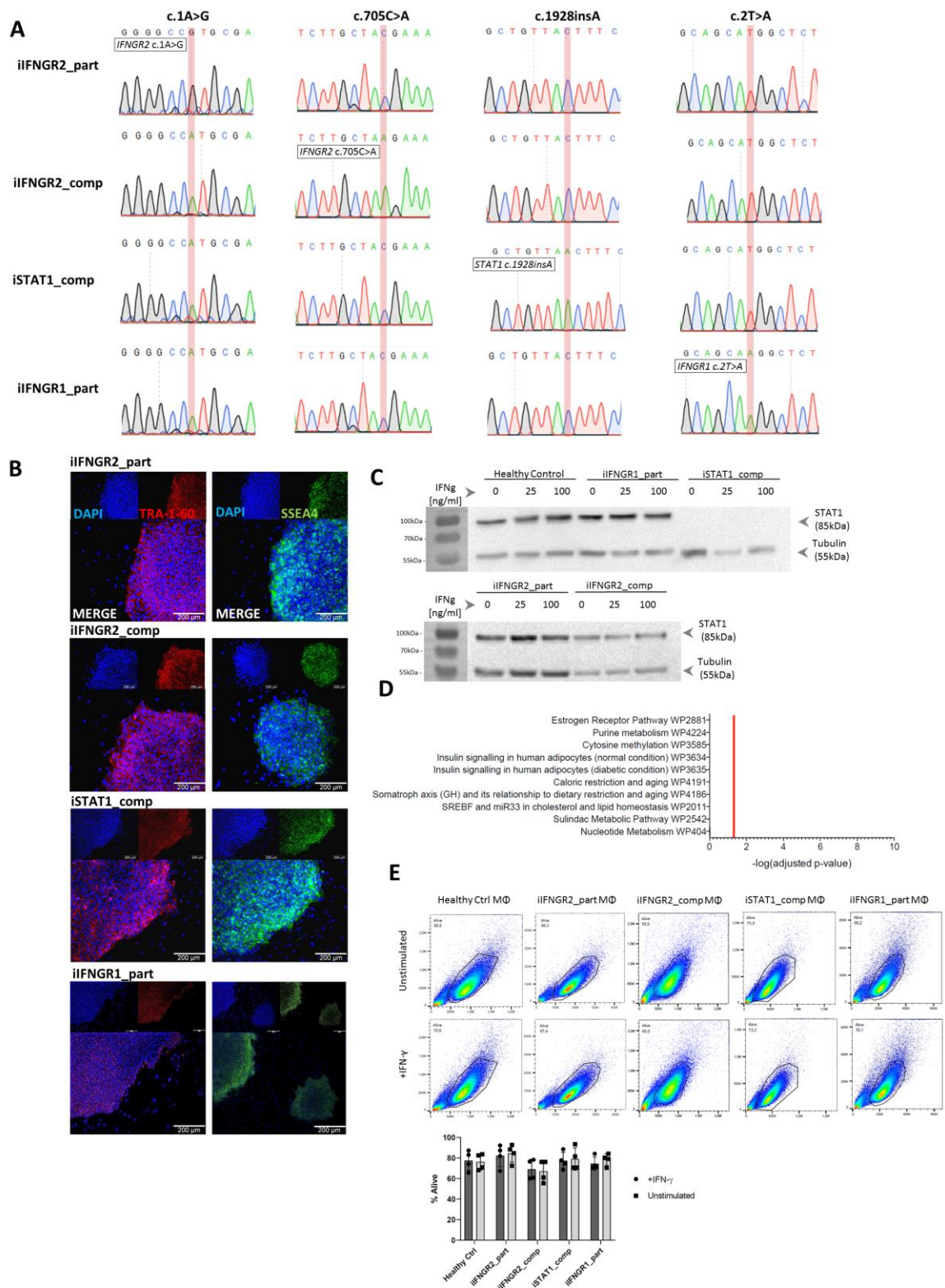

Supplement: Supplementary file 1 [file cells-09-00483-s001.pdf]
